# Prediction and optimization of indirect shoot regeneration of *Passiflora caerulea* using machine learning and optimization algorithms

**DOI:** 10.1186/s12896-023-00796-4

**Published:** 2023-08-01

**Authors:** Marziyeh Jafari, Mohammad Hosein Daneshvar

**Affiliations:** 1 Department of Horticultural Science, College of Agriculture, Shiraz University, Shiraz, 7144113131 Iran; 2 Department of Horticultural Sciences, Agricultural Sciences and Natural Resources University of Khuzestan, Mollasani, 6341773637 Iran

**Keywords:** Artificial intelligence, Callus type, In vitro culture, Micropropagation, Modeling, Passion fruit, Plant growth regulator

## Abstract

**Background:**

Optimization of indirect shoot regeneration protocols is one of the key prerequisites for the development of *Agrobacterium*-mediated genetic transformation and/or genome editing in *Passiflora caerulea*. Comprehensive knowledge of indirect shoot regeneration and optimized protocol can be obtained by the application of a combination of machine learning (ML) and optimization algorithms.

**Materials and methods:**

In the present investigation, the indirect shoot regeneration responses (i.e., *de novo* shoot regeneration rate, the number of *de novo* shoots, and length of *de novo* shoots) of *P. caerulea* were predicted based on different types and concentrations of PGRs (i.e., TDZ, BAP, PUT, KIN, and IBA) as well as callus types (i.e., callus derived from different explants including leaf, node, and internode) using generalized regression neural network (GRNN) and random forest (RF). Moreover, the developed models were integrated into the genetic algorithm (GA) to optimize the concentration of PGRs and callus types for maximizing indirect shoot regeneration responses. Moreover, sensitivity analysis was conducted to assess the importance of each input variable on the studied parameters.

**Results:**

The results showed that both algorithms (RF and GRNN) had high predictive accuracy (R^2^ > 0.86) in both training and testing sets for modeling all studied parameters. Based on the results of optimization process, the highest *de novo* shoot regeneration rate (100%) would be obtained from callus derived from nodal segments cultured in the medium supplemented with 0.77 mg/L BAP plus 2.41 mg/L PUT plus 0.06 mg/L IBA. The results of the sensitivity analysis showed the explant-dependent impact of exogenous application of PGRs on indirect *de novo* shoot regeneration.

**Conclusions:**

A combination of ML (GRNN and RF) and GA can display a forward-thinking aid to optimize and predict in vitro culture systems and consequentially cope with several challenges faced currently in *Passiflora* tissue culture.

**Supplementary Information:**

The online version contains supplementary material available at 10.1186/s12896-023-00796-4.

## Introduction

Passionflower (*Passiflora caerulea* L.) is considered to be one of the most well-known climbing, evergreen shrub species [1]. *P. caerulea* is most often cultivated as a fruit crop, ornamental, or medicinal plant in virtually all tropical and subtropical regions of the world [2]. Due to the unique secondary metabolite profiles and phytochemical compositions of *P. caerulea* oils, there remain certain unexplored applications plant that relate to different fields of research [3, 4]. Various phenols, alkaloids, glycosides, flavonoids, and saponins, represent *P. caerulea* compounds of high medicinal and industrial interest [5]. Improving *P. caerulea* with selected utility traits broadens its biotechnological applicability, which forms the basis of the passionflower industry [6].

The micropropagation procedure, as vegetative reproduction in in vitro cultures, is an excellent way to obtain clones (i.e., plants genetically identical to the parent plants) and genetic improvement through genetic engineering approaches [7]. A new plant arises from the existing meristems of the parent plant, from adventitious meristems [8], or indirectly through the formation of callus (undifferentiated mass of tissue) [9, 10]. Micropropagation represents a common method of germplasm and biodiversity conservation for cultivated, threatened, and endangered species [1]. One of the most important purposes of micropropagation is also obtaining secondary metabolites [11–13]. The micropropagation process is generally carried out in a laboratory setting with controlled light and temperatures, under axenic conditions [14]. Complete isolation from the external environment provides a given plant with protection against potential threats, such as the presence of parasites, viruses, bacteria, or abiotic factors that can negatively influence growth development [15]. The development of an optimal micropropagation protocol makes it possible to obtain regenerated plants with significant healing potential, which are not easily accessible due to the small area of occurrence or are exposed to dangerous factors in the natural environment [16, 17]. One of the most effective in vitro culture methods is indirect shoot regeneration, where callus is used to obtain *de novo* shoots [18, 19].

**Fig. 1:**
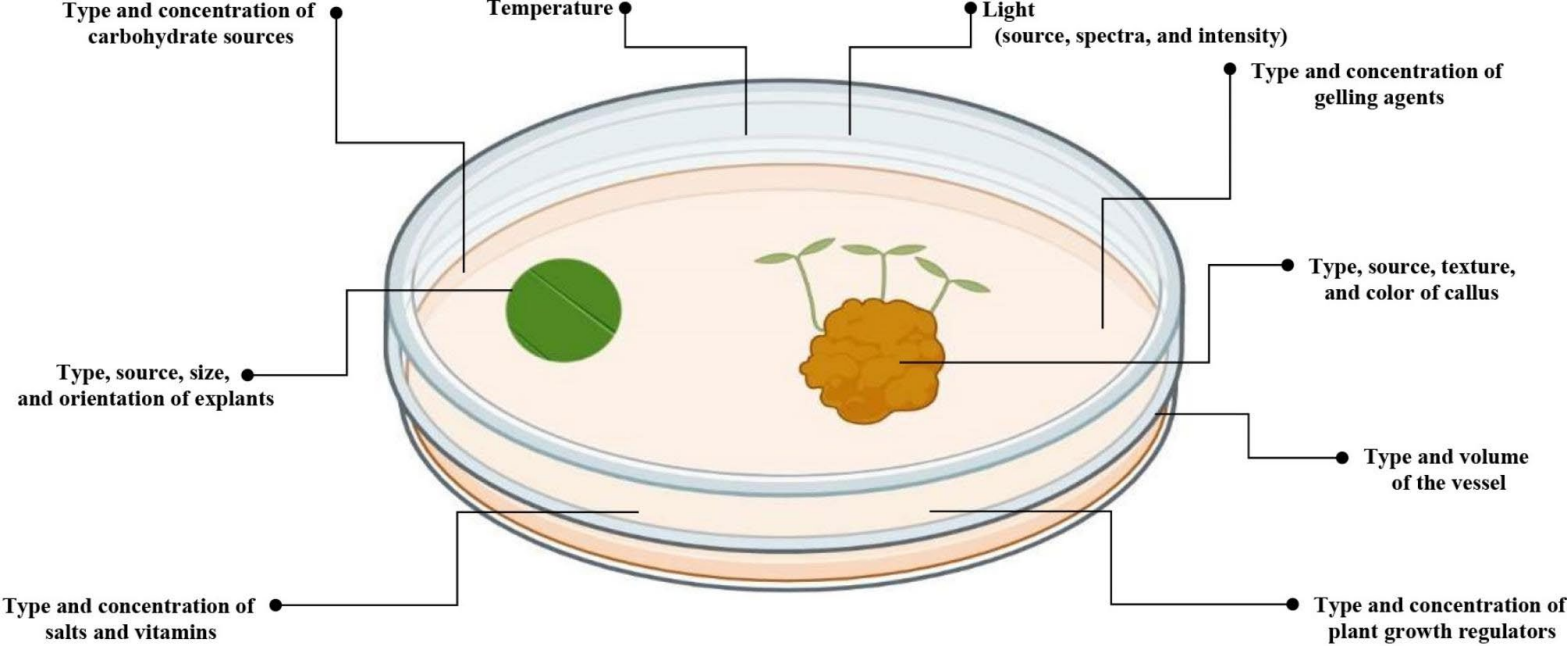
Schematic view of factors influencing indirect *de novo* shoot regeneration

The indirect shoot regeneration protocol can be divided into three basic phases [6]. In phase I, the plant material is selected [20]. This stage is extremely important because improperly selected explants can determine the results of cultivating [21, 22]. The explants should be taken from a young, healthy plant, living in an optimal environment, developing in a favorable period of the year (in spring, plants grow most intensively and are most productive) [23]. The explant should be taken from the part of the plant that has meristematic cells, which guarantees further growth [20, 24, 25]. Sterilization of plant material to be cultured (seed or explant) is critically important to facilitate the axenic integrity of the culture [15]. Sterilization consists of rinsing the material in sodium, calcium or potassium hypochlorite or in ethanol and rinsing three times in sterile water [15]. Then, in phase II, the culture is established. The prepared explants should be transferred to a nutrient medium containing all micro- and macro-elements necessary for the in vitro plant’s growth, as well as appropriate carbohydrate sources and exogenous phytohormones determining the direction of development and influencing the physiological processes of explants [26, 27]. Explant orientation and placement within the culture vessel are also important factors impacting specimen quality. The proximity of explants to one another and proper exposure to media can dramatically influence various developmental characteristics that relate to the integrity of final products [6]. In the initial stage of growth, callus formation can be observed. Phase III consists in extending the cultivation of callus on a medium enriched with phytohormones until the formation of *de novo* shoots is obtained [20]. In this phase, several factors (e.g., type of callus, medium composition, plant growth regulators (PGRs), light, and temperature) are influenced indirect *de novo* shoot regeneration [20, 27–30] (Fig. 1). Though optimization of these factors is necessary for successful indirect shoot regeneration, conventional statistical models are often inadequate and laborious due to manual processing and sequential assessment of single factors [31]. Therefore, novel and innovative computational approaches using machine learning (ML) can be adopted to enhance the analytical and predictive measures required to optimize indirect *de novo* shoot regeneration [32].

Machine learning is defined as an evolving sub-branch of artificial intelligence which can be considered a reliable and promising computational method to predict and optimize a broad range of complicated biological systems [33–49]. Analyzing tissue culture datasets and predicting optimized treatments using ML algorithms represents a favorable approach to in vitro research [32, 36, 50]. Specifically, regression versions of ML algorithms (e.g., generalized regression neural network (GRNN) and random forest (RF)) are currently being applied to several areas of plant tissue culture research [32], including callogenesis [51], shoot proliferation [52], androgenesis [53], somatic embryogenesis [54], and direct shoot regeneration [55].

There is currently no study that enlists ML methods for modeling and optimizing indirect, *de novo* shoot regeneration. The current study represents the first. Since ML methods represent powerful approaches to glean insight about the nature of in vitro biology, this work enlists two ML algorithms (GRNN and RF) to develop a predictive model that relates callus type, PGR type, and PGR concentration to the success of indirect, de novo shoot regeneration of *P. caerulea*.

## Materials and methods

### Plant material and experimental design

Seeds of *P. caerulea* were purchased from the Seed and Plant Improvement Institute, Karaj, Iran. All the experiments done on *P. caerulea* are in compliance with relevant institutional, national, and international guidelines and legislation. The seed sterilization and germination of *P. caerulea* were performed based on our previous protocol [15]. In the current study, three different explants (i.e., leaf, internode, and node) with 0.5 cm lengths were selected from a four-week-old in vitro-grown seedling of *P. caerulea*. In order to develop callus, leaves were cultured in MS medium containing 0.6 g/L agar and 30 g/L sucrose along with 2.0 mg/ L 2,4-Dichlorophenoxyacetic acid (2,4-D) plus 0.2 mg/L indole-3-butyric acid (IBA) on the abaxial side, while internode and node explants were horizontally cultured on the mentioned medium. Cultures were maintained in a growth chamber under dark conditions at 25 °C ± 2 °C for one month, at which point the calli produced was used as explants for the indirect, *de novo* shoot regeneration experiment.

To study the effect of plant growth regulators and different calli (i.e., callus derived from different explants including leaf, node, and internode), MS medium containing 0.6 g/L agar and 30 g/L sucrose was used as a basal medium. The media contained various exogenous plant growth regulators at different concentrations including thidiazuron (TDZ: 0.0, 0.5, and 1.0 mg/L), 6-benzylaminopurine (BAP: 0.0, 0.5, 1.0, 1.5, 2.0 mg/L), kinetin (KIN: 0.0, 0.5, 1.0, 2.0 mg/L), putrescine (PUT: 0.0, 100, 300, 500 mg/L), and IBA (0.0, 0.05, 0.1, 0.15, 0.2 mg/L). The experiment was performed based on a completely randomized design with a total of 39 treatments in triplicate. A list of treatments is presented in Table 1. Each replicate consisted of 10 culture boxes and one callus was cultured in each box. The pH of all the media was adjusted to 5.7 before autoclaving at 121 °C at 0.1 MPa for 20 min. All the chemicals for in vitro culture were supplied by Merck (Sigma-Aldrich products, Irvine, UK). Experimental cultures were maintained in a growth chamber at 25 °C ± 2 °C, 47 ± 3 µmol m^2^ s^− 1^ irradiance for two months, at which point *de novo* shoot regeneration rate, number of *de novo* shoots, and length of *de novo* shoots were measured. The obtained data (Additional file 1) was used as a dataset to feed ML algorithms.

**Table 1:** Effect of plant growth regulators and type of callus on indirect de novo shoot regeneration in P. caerulea

Callus type	BAP (mg/L)	KIN (mg/L)	TDZ (mg/L)	PUT (mg/L)	IBA (mg/L)	Regeneration rate (%)	Shoot number	Shoot length (cm)
Leaf	0	0	0	0	0	0.00 ± 0.000	0.00 ± 0.000	0.00 ± 0.000
Node	0	0	0	0	0	0.00 ± 0.000	0.00 ± 0.000	0.00 ± 0.000
Internode	0	0	0	0	0	0.00 ± 0.000	0.00 ± 0.000	0.00 ± 0.000
Leaf	1	0	0	0	0.1	90.00 ± 0.000	8.33 ± 0.318	2.53 ± 0.145
Node	1	0	0	0	0.1	100.00 ± 0.000	8.87 ± 0.233	2.67 ± 0.176
Internode	1	0	0	0	0.1	90.00 ± 0.000	8.40 ± 0.200	2.23 ± 0.120
Leaf	1.5	0	0	0	0.15	60.00 ± 0.000	6.30 ± 0.208	2.30 ± 0.153
Node	1.5	0	0	0	0.15	73.33 ± 3.333	6.90 ± 0.100	2.73 ± 0.088
Internode	1.5	0	0	0	0.15	60.00 ± 0.000	6.17 ± 0.145	2.20 ± 0.115
Leaf	2	0	0	0	0.2	90.00 ± 0.000	7.67 ± 0.233	2.47 ± 0.203
Node	2	0	0	0	0.2	100.00 ± 0.000	7.97 ± 0.233	2.73 ± 0.186
Internode	2	0	0	0	0.2	86.67 ± 3.333	7.40 ± 0.173	2.53 ± 0.186
Leaf	0	0	0.5	0	0.05	80.00 ± 0.000	8.40 ± 0.252	2.40 ± 0.208
Node	0	0	0.5	0	0.05	93.33 ± 3.333	8.67 ± 0.145	3.00 ± 0.115
Internode	0	0	0.5	0	0.05	83.33 ± 3.333	8.30 ± 0.100	2.20 ± 0.115
Leaf	0	0	1	0	0.1	60.00 ± 0.000	6.33 ± 0.133	1.30 ± 0.173
Node	0	0	1	0	0.1	70.00 ± 0.000	6.43 ± 0.267	1.80 ± 0.115
Internode	0	0	1	0	0.1	56.67 ± 3.333	6.07 ± 0.133	1.17 ± 0.088
Leaf	0	1	0	0	0.1	43.33 ± 3.333	5.40 ± 0.252	1.27 ± 0.145
Node	0	1	0	0	0.1	50.00 ± 0.000	5.57 ± 0.167	1.50 ± 0.153
Internode	0	1	0	0	0.1	43.33 ± 3.333	5.13 ± 0.120	1.13 ± 0.088
Leaf	0	2	0	0	0.2	50.00 ± 0.000	4.73 ± 0.186	1.20 ± 0.115
Node	0	2	0	0	0.2	50.00 ± 0.000	5.23 ± 0.088	1.40 ± 0.208
Internode	0	2	0	0	0.2	46.67 ± 3.333	4.27 ± 0.186	1.13 ± 0.088
Leaf	0	0	0	300	0	30.00 ± 0.000	4.23 ± 0.133	1.23 ± 0.186
Node	0	0	0	300	0	43.33 ± 3.333	4.73 ± 0.133	1.47 ± 0.120
Internode	0	0	0	300	0	30.00 ± 0.000	4.33 ± 0.186	1.27 ± 0.145
Leaf	0	0	0	500	0	23.33 ± 3.333	3.53 ± 0.273	1.27 ± 0.176
Node	0	0	0	500	0	30.00 ± 0.000	4.20 ± 0.000	1.63 ± 0.088
Internode	0	0	0	500	0	23.33 ± 3.333	3.37 ± 0.120	1.30 ± 0.115
Leaf	0.5	0	0	100	0	20.00 ± 0.000	2.70 ± 0.153	0.99 ± 0.007
Node	0.5	0	0	100	0	30.00 ± 0.000	3.30 ± 0.200	1.20 ± 0.115
Internode	0.5	0	0	100	0	20.00 ± 0.000	2.33 ± 0.233	0.83 ± 0.088
Leaf	0	0	0.5	100	0	10.00 ± 0.000	1.87 ± 0.067	0.93 ± 0.145
Node	0	0	0.5	100	0	20.00 ± 0.000	2.47 ± 0.067	1.10 ± 0.115
Internode	0	0	0.5	100	0	10.00 ± 0.000	1.43 ± 0.133	0.87 ± 0.088
Leaf	0.5	0.5	0.5	0	0	10.00 ± 0.000	1.60 ± 0.100	0.93 ± 0.145
Node	0.5	0.5	0.5	0	0	16.67 ± 3.333	2.33 ± 0.133	1.33 ± 0.176
Internode	0.5	0.5	0.5	0	0	10.00 ± 0.000	1.40 ± 0.100	0.83 ± 0.120

BAP: 6-benzylaminopurine; IBA: indole-3-butyric acid; KIN: kinetin; PUT: putrescine; TDZ: thidiazuron. Values represent mean ± standard error.

### Machine learning procedures

**Fig. 2:**
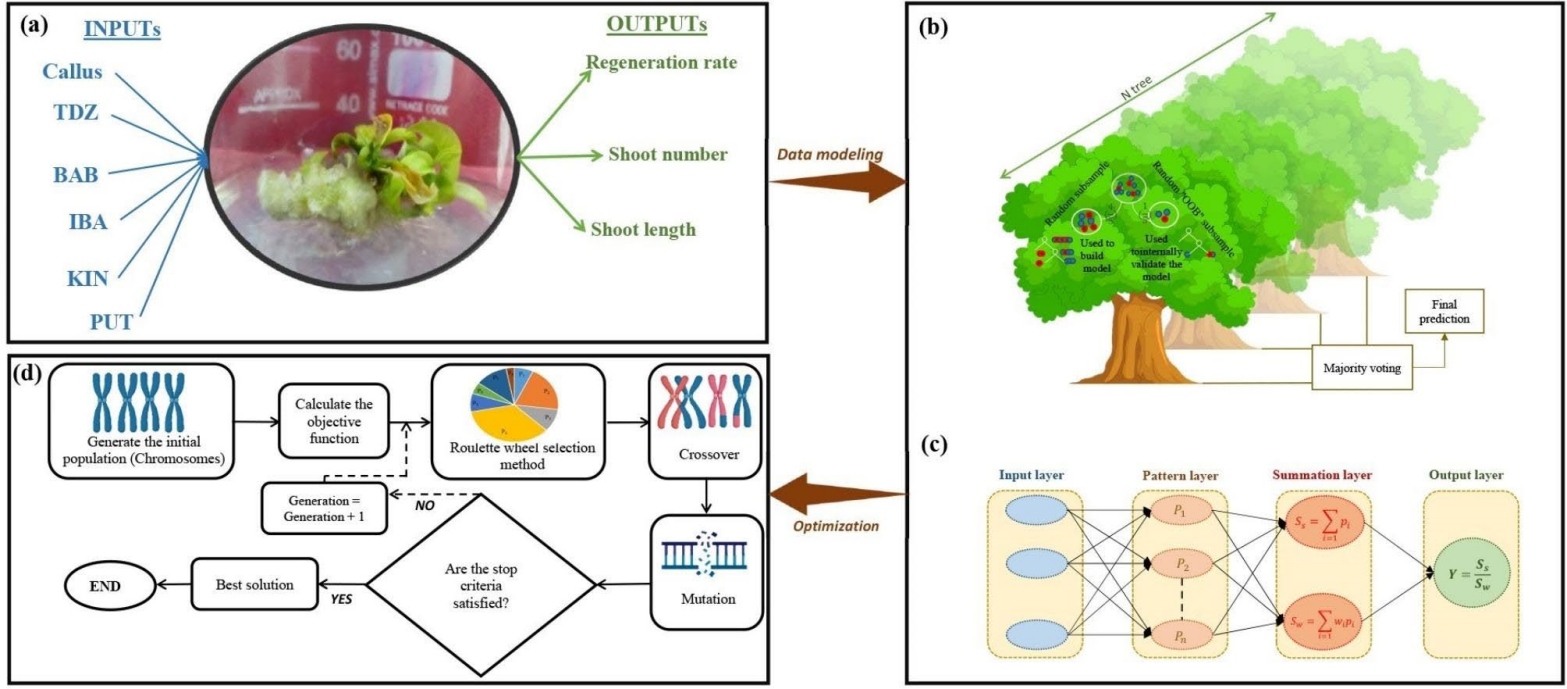
The schematic representation of the step-by-step methodology of the current study including **(a)** dataset consists of inputs (i.e., callus type, 6-benzylaminopurine (BAP), indole-3-butyric acid (IBA), kinetin (KIN), putrescine (PUT), and thidiazuron (TDZ)) and outputs (i.e., regeneration rate, shoot number, and shoot length), **(b, c)**, data modeling through generalized regression neural network (GRNN) and random forest (RF), respectively, and **(d)** optimization process through a genetic algorithm (GA).

Before using ML algorithms, the data was normalized by using Box-Cox transformation. Although principal component analysis (PCA) was applied to determine outliers, no outliers were detected in the dataset. Type of callus (i.e., callus derived from different explants including leaf, node, and internode), TDZ, BAP, PUT, KIN, and IBA were considered as input variables, while *de novo* shoot regeneration rate, number of *de novo* shoots, and length of *de novo* shoots were fed to ML as target variables (Fig. 2a). Moreover, 80% and 20% of the dataset were randomly selected to train and test ML algorithms. In the current investigation, two supervised ML algorithms (RF and GRNN) were used to model and predict the indirect *de novo* shoot regeneration of *P. caerulea*.

The regression version of the RF algorithm (Fig. 2b) uses different subsets of training data by randomly resampling the main dataset with the substitution for generating several *T* of regression trees. Moreover, the RF algorithm, during induction of tree growth, uses the best predictor among a predictor subset (*p*) that has been randomly selected from all input predictors. Therefore, the correlation of the different regression trees is avoided which leads to higher prediction accuracy. Finally, all *T* regression trees are averaged to obtain the best final prediction (Fig. 2b). GRNN as one of the sub-branches of artificial neural networks (ANNs) consisting of four layers (i.e., input, pattern, summation, and output) was used as another supervised ML algorithm in the current study (Fig. 2c). GRNN is based on a radial basis network which calculates the final prediction based on the average of all the weighted observed output data of former layers (Fig. 2c).

The accuracy and efficiency of the ML algorithms (FR and GRNN) were evaluated and compared by using three different performance criteria including coefficient of determination (R^2^), mean absolute error (MAE), and root mean square error (RMSE).

### Optimization process

In the current study, a genetic algorithm (GA) was used to find the optimal level of TDZ, BAP, PUT, KIN, IBA, and callus type in order to maximize *de novo* shoot regeneration rate, number of *de novo* shoots, and length of *de novo* shoots. Hence, the developed ML models were fed to GA (Fig. 2d) where the generation number, initial population, selection function, cross-over function, crossover rate, mutation function, and mutation rate were respectively considered as 1000, 200, Roulette Wheel, two-point cross-over, 0.6, uniform, and 0.05.

### Sensitivity analysis

Sensitivity analysis was conducted to evaluate the importance degree of callus, TDZ, BAP, PUT, KIN, and IBA on *de novo* shoot regeneration rate, number of *de novo* shoots, and length of *de novo* shoots by calculating variable sensitivity error (VSE) and variable sensitivity ratio (VSR). VSE shows the RMSE of the developed ML model (i.e., GRNN) when the input is eliminated from the developed model. VSR equals the ratio of VSE and RMSE of the developed ML when all inputs are available. Then, the importance of input variables is ranked based on the value of VSR. All the analyses were also conducted using MATLAB® software.

## Results

### Effect of plant growth regulators and type of callus on indirect ***de novo*** shoot regeneration in ***P. caerulea***

In the current study, the effect of different types and concentrations of PGRs (i.e., TDZ, BAP, PUT, KIN, and IBA) as well as callus type (i.e., callus derived from different explants including leaf, node, and internode) were evaluated on indirect shoot regeneration responses (i.e., *de novo* shoot regeneration rate, number of *de novo* shoots, and length of *de novo* shoots) of *P. caerulea*. Based on Table 1, different indirect shoot regeneration responses were obtained from different types of calli in the media containing various combinations of PGRs. The highest *de novo* shoot regeneration rate, the number of *de novo* shoots, and the length of *de novo* shoots were obtained from callus derived from node segment followed by calli derived from leaf and internode explants (Table 1). In relation to the combination of PGRs, the media containing 1 mg/L BAP along with 0.1 mg/L IBA led to the maximum *de novo* shoot regeneration rate and the number of *de novo* shoots, while the highest length of *de novo* shoots was observed in the media consisting of 0.5 mg/L TDZ along with 0.05 mg/L IBA (Table 1). Also, our results showed that there was no *de novo* shoot regeneration in the media without PGRs (Table 1).

In relation to the interaction between callus type and PGRs, the maximum *de novo* shoot regeneration rate (100 ± 0.0%) and number of *de novo* shoots (8.87 ± 0.233) were observed in calli derived from nodal segments cultured in the media containing 1 mg/L BAP along with 0.1 mg/L IBA (Table 1). Moreover, the highest length of *de novo* shoot (3 ± 0.115 cm) was observed in calli derived from nodal segments cultured in the media containing 0.5 mg/L TDZ along with 0.05 mg/L IBA (Table 1).

### Evaluation of generalized regression neural network (GRNN) and random forest (RF)

**Fig. 3:**
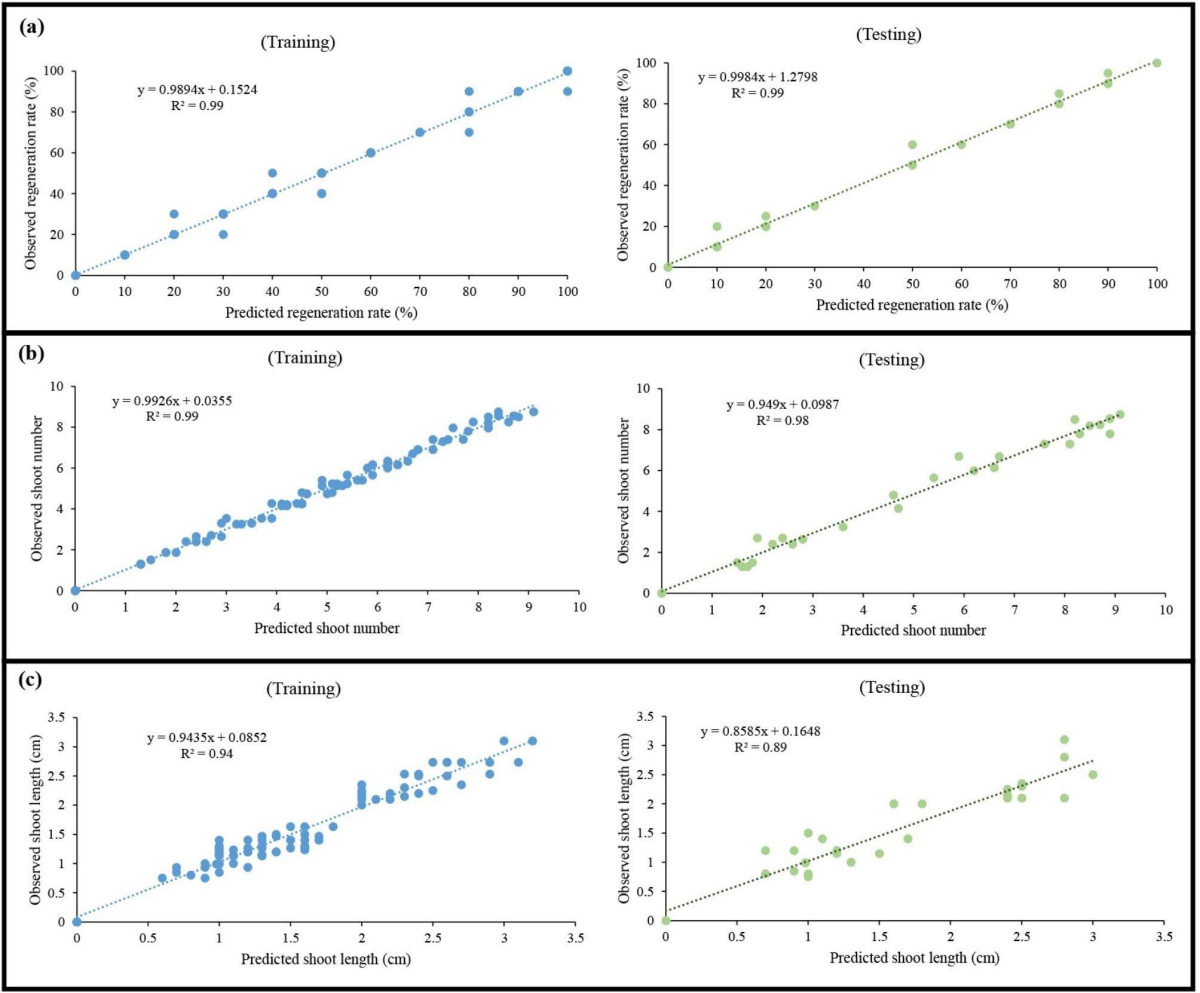
Scatter plot of values of observations vs. predictions in training sets and testing sets of generalized regression neural network (GRNN) in **(a)** regeneration rate, **(b)** shoot number, and **(c)** shoot length

In the present investigation, the indirect shoot regeneration responses (i.e., *de novo* shoot regeneration rate, the number of *de novo* shoots, and length of *de novo* shoots) of *P. caerulea* were predicted based on different types and concentrations of PGRs (i.e., TDZ, BAP, PUT, KIN, and IBA) as well as callus types (i.e., callus derived from different explants including leaf, node, and internode) using GRNN and RF algorithms. Based on the results (Table 2), the GRNN algorithm led to the development of predictive models with higher R^2^ in both testing and training subsets in comparison to RF for all indirect shoot regeneration responses including *de novo* shoot regeneration rate (R^2^ > 0.99 for GRNN vs. R^2^ > 0.96 RF), the number of *de novo* shoots (R^2^ > 0.98 for GRNN vs. R^2^ > 0.97 for RF), and length of *de novo* shoots (R^2^ > 0.89 for GRNN vs. R^2^ > 0.86 for RF). Furthermore, the observed and predicted values in all indirect shoot regeneration responses were perfectly correlated in both training and testing subsets (Fig. 3).

**Table 2:** Performance criteria of machine learning algorithms for indirect de novo shoot regeneration of P. caerulea in training and testing subsets

Output	ML Model	subset	R^2^	RMSE	MAE
Regeneration rate	GRNN	Training	0.99	2.65	0.00
		Testing	0.99	3.08	1.21
	RF	Training	0.97	3.02	0.29
		Testing	0.96	3.12	1.45
Shoot number	GRNN	Training	0.99	0.21	0.00
		Testing	0.98	0.43	0.14
	RF	Training	0.98	0.45	0.02
		Testing	0.97	0.63	0.25
Shoot length	GRNN	Training	0.94	0.18	0.00
		Testing	0.89	0.31	0.07
	RF	Training	0.91	0.25	0.04
		Testing	0.86	0.43	0.12

GRNN: generalized regression neural network; MAE: mean absolute error; ML; machine learning; R^2^: coefficient of determination; RF: random forest; RMSE: root mean square error.

In addition, RMSE was used to evaluate and compare the accuracy of algorithms (i.e., GRNN and RF). The results showed that the GRNN algorithm led to higher accuracy and performance in either testing or training subsets in comparison to RF for all indirect shoot regeneration responses including *de novo* shoot regeneration rate (RMSE < 3.08 for GRNN vs. RMSE < 3.12 for RF), the number of *de novo* shoots (RMSE < 0.43 for GRNN vs. RMSE < 0.63 for RF), and length of *de novo* shoots (RMSE < 0.31 for GRNN vs. RMSE < 0.43 for RF) (Table 2). MAE as another performance criterion showed that the GRNN algorithm led to higher accuracy and performance in either testing or training subsets in comparison to RF for all indirect shoot regeneration responses including *de novo* shoot regeneration rate (MAE < 1.21 for GRNN vs. MAE < 1.45 for RF), the number of *de novo* shoots (MAE < 0.14 for GRNN vs. MAE < 0.25 for RF), and length of *de novo* shoots (MAE < 0.07 for GRNN vs. MAE < 0.12 for RF) (Table 2).

### Optimization process

The developed GRNN models (the most accurate algorithm in the current investigation) were integrated into the genetic algorithm (GA) as a single-objective evolutionary optimization method to optimize the concentration of PGRs (i.e., TDZ, BAP, PUT, KIN, and IBA) and callus types (i.e., callus derived from different explants including leaf, node, and internode) for maximizing indirect shoot regeneration responses (i.e., *de novo* shoot regeneration rate, the number of *de novo* shoots, and length of *de novo* shoots). Based on the results of optimization using GRNN-GA (Table 3), the highest *de novo* shoot regeneration rate (100%) would be obtained from callus derived from nodal segments cultured in the medium supplemented with 0.77 mg/L BAP plus 2.41 mg/L PUT plus 0.06 mg/L IBA. Also, the maximum number of shoots (8.75) would be obtained from callus derived from nodal segments cultured in the medium supplemented with 0.76 mg/L BAP plus 0.005 mg/L TDZ plus 0.96 mg/L PUT plus 0.076 mg/L IBA (Table 3). Moreover, the highest length of shoot (3.1 cm) would be obtained from callus derived from nodal segments cultured in the medium supplemented with 0.002 mg/L BAP plus 0.007 mg/L KIN plus 0.5 mg/L TDZ plus 1.006 mg/L PUT plus 0.17 mg/L IBA (Table 3).

**Table 3:** Determination of the optimal level of plant growth regulators and callus types for maximizing indirect shoot regeneration responses through genetic algorithm

Fitness function	Callus type	BAP (mg/L)	KIN (mg/L)	TDZ (mg/L)	PUT (mg/L)	IBA (mg/L)	Predicted-optimized outcome
Regeneration rate (%)	Node	0.77	0.00	0.00	2.41	0.06	100
Shoot number	Node	0.76	0.00	0.00	0.96	0.08	8.75
Shoot length (cm)	Node	0.002	0.007	0.500	1.006	0.172	3.100

BAP: 6-benzylaminopurine; IBA: indole-3-butyric acid; KIN: kinetin; PUT: putrescine; TDZ: thidiazuron.

### Importance degree of each input on ***P. caerulea*** indirect shoot regeneration responses

In the current study, sensitivity analysis through the calculation of variable sensitivity ratio (VSR) was conducted to assess the importance of each input variable (i.e., callus type, TDZ, BAP, PUT, KIN, and IBA) on the studied objective functions (i.e., *de novo* shoot regeneration rate, the number of *de novo* shoots, and length of *de novo* shoots). According to our results (Table 4), the callus type was the most important factor for indirect shoot regeneration rate followed by BAP, IBA, PUT, TDZ, and KIN respectively. Callus type > BAP > KIN > TDZ > IBA > PUT was ranked for number of shoots (Table 4). In addition, callus type > BAP > KIN > PUT > TDZ > IBA was ranked for shoot length (Table 4). VSR values for callus type are considerably higher than all PGRs (Table 4), indicating callus type to be the principal factor impacting indirect, *de novo* shoot regeneration. This emphasizes the explant-dependent impact of exogenous PGRs on indirect, *de novo* shoot regeneration.

**Table 4:** Importance degree of each input on P. caerulea indirect shoot regeneration responses through sensitivity analysis

Outcome	Item	Subset	Callus type	BAP	KIN	TDZ	PUT	IBA
Regeneration rate	VSR	Training	3.11	2.11	1.12	1.15	1.21	1.31
		Testing	2.34	1.54	0.86	1.00	1.00	1.02
	Rank		1	2	6	5	4	3
Shoot number	VSR	Training	4.97	1.94	1.49	1.37	1.20	1.25
		Testing	2.41	0.90	0.88	0.86	0.78	0.83
	Rank		1	2	3	4	6	5
Shoot length	VSR	Training	2.33	1.59	1.46	1.34	1.42	1.22
		Testing	1.41	0.94	0.91	0.73	0.86	0.73
	Rank		1	2	3	5	4	6

BAP: 6-benzylaminopurine; IBA: indole-3-butyric acid; KIN: kinetin; PUT: putrescine; TDZ: thidiazuron; VSR: variable sensitivity ratio.

## Discussion

Indirect shoot regeneration of *P. caerulea* can be applied to production of secondary metabolites, clonal production, and gene bank establishment [6, 28]. The latter two of which are integral to genotype preservation, while the former has broad biotechnological and medicinal applications. However, it is necessary to optimize several factors involved in *de novo* soot regeneration from callus cultures [32]. PGR type and concentration, in addition to the origin of calli represent fundamental factors affecting indirect, *de novo* shoot regeneration [27]. Importantly, the interaction of PGRs and callus type represents a critical factor impacting success of this process, which was exemplified in our results. In fact, any given concentration of PGRs will fall within the various dose-response range according to the species and origin of the calli [6]. Therefore, the concentration of PGRs should be optimized before their application. However, constructing and optimizing tissue culture protocols represents a major challenge to the field as a whole [51]. Conventional statistical methods and large experiments involving thousands of treatments have traditionally been employed to develop tissue culture protocols [56]. Such techniques can only assess simple linear/curvilinear relationships between variables by serially assessing the influence of individual factors without accounting for dynamic, interactional effects of these factors on in vitro plant growth and development [56]. Additionally, traditional statistical methods and associated experimental systems are largely constrained by the extensive footprint of treatments and replications required for accurate data modeling [56]. Ultimately, such approaches can take insurmountable timespans and resources to develop improved, tough suboptimal tissue culture protocols [36]. Thus, due to the potential to exclude dynamic interactional effects of combined factors, optimization methods must be re-imagined using a modern approach to simultaneously optimize multiple factors for development of precision techniques [57]. For these reasons, applying new powerful approaches for analyzing and predicting in vitro culture systems is crucial [32].

Using modern computational approaches, ML offers a more simple and reliable approach to recognize and diagnose complex datasets that are commonly obtained from tissue culture experiments [32]. The powerful interoperative processes of newly developed nonlinear machine learning algorithms have recently been a focus for plant system biology [38], plant breeding [33], and plant tissue culture [32]. These methods remove uncertainties associated with dynamic tissue responses by diagnosing complex patterns and uses algorithms to predict optimal combinations of factors for desired results [36]. These patterns can then be analyzed using optimization algorithms to predict optimal combinations of factors for desired outcomes [56]. The robustness and accuracy of hybrid ML-optimization algorithms in modeling and predicting different in vitro culture systems have been previously confirmed in different species such as chrysanthemum [54, 58–62], passion fruit [31], *Prunus* rootstock [63–65], hazel [66], tomato [53], chickpea [52, 67], wheat [68], cannabis [56, 57, 69–72], and ajowan [73].

Therefore, in the current study, two ML algorithms (GRNN and RF) were employed to develop a predictive model for getting in-depth insight into the effect of PGRs (i.e., TDZ, BAP, PUT, KIN, and IBA) and callus types (i.e., callus derived from different explants including leaf, node, and internode) on indirect *de novo* shoot regeneration of *P. caerulea*. Our results showed that both RF and GRNN could be accurately model and predict indirect *de novo* shoot regeneration. In line with our results, previous studies have shown that GRNN is a powerful ML algorithm for modeling and predicting different plant biological systems such as seed germination [71], in vitro shoot regeneration [59], shoot growth and development [56], in vitro sterilization [69], secondary metabolite production [66], in vitro rooting [31], and morphological response of the aboveground parts of the plant to drought stress [74]. Moreover, the accuracy of RF has been previously demonstrated in different areas of plant science such as plant tissue culture [70], breeding [33], high-throughput phenotyping [41], and gRNA designing for CRISPR-based methods [72]. Generally, the results of the current study showed that ML is a reliable and accurate approach for predicting indirect *de novo* shoot regeneration.

Based on the result of sensitivity analysis, callus type was the most important factor for all the indirect regeneration parameters, followed by PGRs (i.e., BAP, IBA, PUT, TDZ, and KIN for indirect shoot regeneration rate; BAP, KIN, TDZ, IBA, and PUT for number of shoots; BAP, KIN, PUT, TDZ, and IBA for shoot length). It is well-documented that the callus type plays a key role in indirect *de novo* shoot regeneration [1, 6, 28]. Indeed, the various in vitro responses of each type of callus might be due to the differences in epigenetic regulation as well as endogenous sugars and phytohormones [75]. Similar to our results, previous studies demonstrate that callus type represents the most important factor influencing successful indirect, *de novo* shoot regeneration [6, 27, 28]. Due to the totipotent potential of callus cells, the manipulation of the concentration and ratio of PGRs leads to the differentiation of the callus cells that can ultimately result in *de novo* shoot regeneration [12]. Our results revealed that BAP was the second most important factor in indirect *de novo* shoot regeneration. In line with our results, previous studies showed that BAP led to a higher frequency of regeneration compared to other cytokinins in different *Passiflora* sp. such as *P. trifasciata* [76], *P. foetida* [76, 77], *P. suberosa* [27, 78], *P. caerulea* [79], *P. cincinnata* [80], and *P. cristalina* [81].

The results of the optimization process (GA) showed that the maximum *de novo* shoot regeneration rate would be achieved from callus derived from nodal segments cultured in the medium supplemented with 0.77 mg/L BAP plus 2.41 mg/L PUT plus 0.06 mg/L IBA. The result highlighted the importance of balances among PGRs, especially between cytokinins and auxins. In general, a low concentration of auxin and a high concentration of cytokinins induces *de novo* shoot regeneration [6, 12]. In line with our results, Rosa et al. [27] reported that a high concentration of cytokinin (BAP) without or with a low concentration of auxin was the best PGRs balance for indirect shoot regeneration in *P. suberosa*. The application of GA in optimizing plant tissue culture processes offers substantial benefits and enhances the reliability of achieving optimal outcomes [82]. GA, a robust optimization technique inspired by natural selection and genetics, proves invaluable in exploring complex solution spaces and identifying optimal configurations [63]. In the realm of plant tissue culture, GA proves particularly useful in fine-tuning critical parameters, including growth media composition, hormone concentrations, and culture conditions, to maximize desired outcomes such as callogenesis, organogenesis, rhizogenesis, and embryogenesis [29, 31, 56, 63–65, 68, 70]. GA can significantly reduce reliance on time-consuming and expensive trial-and-error experiments [32]. The algorithm’s ability to intelligently evolve and refine solutions based on fitness evaluations not only expedites the optimization process but also ensures more consistent and reliable results [83]. Consequently, GA empowers researchers and plant tissue culture practitioners to efficiently design and implement effective protocols, leading to enhanced plant propagation techniques and expanded biotechnological applications [36]. While previous studies have demonstrated the reliability of GA in optimizing in vitro culture processes [29, 31, 56, 63–65, 68, 70], it is crucial to conduct future research to validate the predicted-optimized (GRNN-GA) results obtained in the current study.

## Conclusion

Optimization of indirect *de novo* shoot regeneration protocols is one of the key prerequisites for the development of *Agrobacterium*-mediated genetic transformation and/or genome editing in *P. caerulea*. Comprehensive knowledge related to indirect shoot regeneration leading to protocol optimization can be achieved by applying the combined ML -optimization algorithm approach. Our results showed that indirect shoot regeneration of *P. caerulea* could be precisely predicted and optimized using methods that link ML (i.e., GRNN and RF) to evolutionary optimization algorithms (i.e., GA). The optimized PGRs and the suitability of the developed model (GRNN-GA) in indirect shoot regeneration should be assessed by future studies in other *Passiflora* species. Moreover, the adaptation of a combination of ML (GRNN and RF) and GA can display a forward-thinking aid to optimize and predict in vitro culture systems and consequentially cope with several challenges faced currently in *Passiflora in vitro* culture.

## Electronic supplementary material

Below is the link to the electronic supplementary material.

Supplementary Material 1

## Data Availability

All data generated or analyzed during this study are included in this published article and its supplementary information files.

## List of the abbreviations

2,4-D: 2,4-Dichlorophenoxyacetic acid
ANN: Artificial neural network
BAP: 6-benzylaminopurine
GA: Genetic algorithm
GRNN: Generalized regression neural network
IBA: Indole-3-butyric acid
KIN: Kinetin
MAE: Mean absolute error
ML: Machine learning
PGR: Plant growth regulator
PUT: Putrescine
R^2^: Coefficient of determination
RF: Random forest
RMSE: Root mean square error
TDZ: Thidiazuron
VSE: Variable sensitivity error
VSR: Variable sensitivity ratio
